# *Aspergillus* Species Causing Invasive Fungal Disease in Queensland, Australia

**DOI:** 10.1007/s11046-023-00713-5

**Published:** 2023-04-17

**Authors:** Adam G. Stewart, Burcu Isler, Peter Simos, Drew Farquhar, Narelle George, Mila Golmayo, Claire Heney

**Affiliations:** 1 Centre for Clinical Research, Faculty of Medicine, The University of Queensland, Royal Brisbane and Women’s Hospital Campus, Brisbane, Australia; 2 Department of Infectious Diseases, Royal Brisbane and Women’s Hospital, Brisbane, Australia; 3 Central Microbiology, Pathology Queensland, Brisbane, Australia; 4 Infection Management Services, Princess Alexandra Hospital, Brisbane, QLD Australia

**Keywords:** *Aspergillus* species, Antifungal susceptibility testing, Azole resistance

## Abstract

**Background:**

*Aspergillus* species are important causes of invasive fungal disease, particularly among those with an impaired immune system. Increasing reports have revealed a rising incidence of antifungal drug resistance among *Aspergillus* spp., particularly among cryptic species. Understanding local antifungal susceptibility patterns is paramount to delivering optimal clinical care.

**Methods:**

*Aspergillus* spp. recovered from clinical specimens between 2000 and 2021 from Pathology Queensland were collected. *Aspergillus* spp. were identified routinely morphologically, and where there was ambiguity or a lack of sporulation, by sequencing of the internal transcribed spacer (ITS) region. All *Aspergillus* spp. that underwent antifungal susceptibility testing according to the CLSI M38-A3 method and were recorded and included in the study. Amphotericin B, voriconazole, posaconazole, isavuconazole, micafungin, caspofungin, and anidulafungin were tested. Pathology Queensland services all public healthcare facilities in Queensland, Australia.

**Results:**

236 *Aspergillus* spp. were identified from clinical specimens during the study period. The most frequent species identified were *Aspergillus* section Fumigati (*n* = 119), *Aspergillus* section Flavi (*n* = 35), *Aspergillus terreus* (*n* = 32) and *Aspergillus niger* (*n* = 29). Overall, MIC_50/90_ values for voriconazole, posaconazole, itraconazole, and isavuconazole were 0.25/1, 0.25/0.5, 0.25/0.5, and 0.5/2 mg/L respectively. Echinocandins demonstrated low MIC values overall with micafungin and anidulafungin both having an MIC_50/90_ of 0.015/0.03 mg/L. A total of 15 cryptic species were identified; high triazole MIC values were observed with a voriconazole MIC_50/90_ of 2/8 mg/L. From 2017 to 2021 we observed an increase in incidence of isolates with high voriconazole MIC values. There was no difference in voriconazole MIC values between *Aspergillus* spp. acquired in North Queensland when compared to Southeast Queensland, Australia.

**Conclusion:**

Increasing reports of antifungal resistance among *Aspergillus* spp. is concerning and warrants further investigation both locally and worldwide. Active surveillance of both the emergence of different *Aspergillus* spp. and changes in antifungal susceptibility patterns over time is crucial to informing clinicians and treatment guidelines.

## Introduction

Invasive aspergillosis (IA) is associated with increased mortality in immunocompromised persons. Patients with acute leukaemia and allogeneic stem cell transplantation comprise the largest risk group, with mortality rates reaching 45% in this population [1]. There has been a steady increase in the incidence of IA (i.e., 4.4% per year per 100,000 persons) over the years, particularly among those with haematological malignancies, solid organ transplant recipients, patients with solid tumours, or chronic renal failure [2].

Azole antifungals are the mainstay of treatment and prophylaxis of IA and have been widely used for these indications [3, 4]. In accordance with widespread clinical and environmental azole use, azole resistant *Aspergillus* spp. are increasingly reported worldwide, both for the azole-naïve and azole-experienced populations, with a prevalence of 3–4% in *Aspergillus* section Fumigati isolates [5–8]. Mortality rates can surpass 80% for infections due to azole-resistant *Aspergillus* spp. [9]. International guidelines recommend against azole monotherapy for detected azole resistance and for the settings where *Aspergillus* spp. azole resistance exceeds 10%; less favourable therapeutic recommendations such as amphotericin monotherapy or echinocandin plus azole combination therapy have been proposed [3, 4, 10]. Implementation of this approach for the management of acute IA requires close surveillance of antifungal resistance at a local, regional, and global level. This is hampered by the lack of availability of routine antifungal susceptibility testing (AFST) in most clinical microbiology laboratories due to the challenges associated with AFST [11].

In addition, recently there has been an emergence of cryptic *Aspergillus* species as a cause of invasive disease. These species are difficult to differentiate from other related species by phenotypic and morphologic features alone. Molecular methods for accurate identification have been developed which include targeted sequencing of genetic markers including the internal transcribed spacer (*ITS*) regions of the rRNA, a portion of the beta-tubulin gene (*BenA*), a fragment of the calmodulin gene (*CaM*), and part of the RNA polymerase II second largest subunit gene (*RPB2*)[12]. Whole genome sequencing (WGS) approaches have also been used for precise species identification [13].

Queensland Health provides a range of serves including haematopoietic stem cell and solid organ transplantation, haematology, medical oncology, intensive care, major surgical sub-specialties, and burns. Queensland is a large state with diverse geography that includes subtropical and tropical coastal regions and inland dry desert areas. Approximately two thirds of the population are concentrated around the Greater Brisbane, Gold Coast, and Sunshine Coast areas in the Southeast corner of the state. For the purposes of this study, we defined North Queensland as a latitude including and above 23.4300° S (Rockhampton Region), and Southeast Queensland as locations below this latitude.

In this study, we aim to characterize *Aspergillus* spp. causing invasive fungal disease in Queensland, Australia, over a 20-year period. This includes the description of the antifungal resistance profile, with an emphasis on azole resistance, thereby demonstrating antifungal resistance trends over time. We will also determine the effect of geography (e.g. tropical and subtropical climate) on antifungal resistance patterns.

## Methods

*Aspergillus* spp. recovered from clinical specimens between 2000 and 2021 from Pathology Queensland Laboratories were identified. Isolates recovered from sterile material and not deemed a contaminant are always sent for susceptibility testing. Other isolates typically require host factors (e.g. haematological malignancy) and/or clinical features (e.g. dense, well circumscribed lesions on computed tomography [CT] chest) to be noted by the Clinical Microbiologist, after consultation with the treating clinician, in order to justify susceptibility testing. Clinical specimen type included tissue (*n* = 81), bronchoalveolar lavage fluid (*n* = 65), upper respiratory tract specimens (*n* = 47), intra-operative or deep swab (*n* = 26), and sterile site fluid (*n* = 17). These samples were set up for fungal culture using a Sabouraud Dextrose Agar (SDA) supplemented with chloramphenicol and gentamicin, which was incubated at 28 degrees Celsius in air for four weeks. *Aspergillus* spp. were initially identified using phenotypic and morphologic methods, which included growth at different temperatures and morphological characteristics on lactophenol cotton blue staining. From 2011, any isolate thought to be clinically significant (as determined by the clinical microbiologist), and that required identification (e.g. failure to sporulate) or AFST, would undergo Sanger sequencing of the ITS region at a reference laboratory. Prior to 2011, the laboratory relied solely on phenotypic and morphologic methods for identification of moulds. Briefly, fungal DNA was extracted using the QIAGEN DNease® UltraClean® Microbial Kit with the addition of Lyticase to the PowerBead tube containing PowerBead and SL solution. Amplicon sequence was undertaken using a 579 base pair fragment of the ITS using the primary pairs P-ITS-1 and P-ITS-4. Analysis using BLASTNR, MycoBank and MycologyLab were utilised.

All *Aspergillus* spp. that underwent AFST were recorded and included in the study. Pathology Queensland does not perform AFST on mould isolates, which are sent to the Australian Mycology Reference Laboratory in Adelaide, South Australia for AFST on a case-by-case basis. Australian laboratories that perform AFST rely on commercial methods; as such, the Sensititre YeastOne (ThermoFisher) was used for *Aspergillus* spp. susceptibility testing [14]. Inoculum suspensions of *Aspergillus* spp. were prepared according to the CLSI M38-A3 document and *A. flavus* ATCC 204,304 and *A. fumigatus* ATCC MYA-3626 were used as CLSI reference strains. Amphotericin B (range, 0.12 to 8 mg/L), voriconazole (range, 0.008 to 8 mg/L), posaconazole (range, 0.008 to 8 mg/L), isavuconazole (range, 0.008 to 8 mg/L), micafungin (range, 0.008 to 8 mg/L), caspofungin (range, 0.008 to 8 mg/L), and anidulafungin (range, 0.015 to 8 mg/L) were tested and recorded. CLSI interpretive criteria were used for susceptibility categorization into wild-type and non-wild-type isolates (CLSI M59 document).

## Results

Overall, 236 *Aspergillus* spp. isolates identified from clinical specimens in patients with suspected invasive *Aspergillus* spp. infection underwent susceptibility testing (Table 1). The most frequent species identified were *Aspergillus* section Fumigati (*n* = 119), *Aspergillus* section Flavi (*n* = 36), *Aspergillus terreus* (*n* = 32), and *Aspergillus niger* (*n* = 29). Other uncommon species identified (*n* = 20) included *A. nidulans, A. calidoustus, A. sydowii, A. ochraceus, A. viridinutans, A. insuetus, A. unguis, A. ustus, A. wisconsinensis,* and *A. lentulus*,. These isolates were identified using molecular techniques. For pooled *Aspergillus* spp., MIC_50/90_ values for voriconazole, posaconazole, itraconazole, and isavuconazole were 0.25/1, 0.25/0.5, 0.25/0.5, and 0.5/2 mg/L respectively. Azole antifungals tested against *Aspergillus* section Fumigati demonstrated favourable in vitro activity overall, with voriconazole and itraconazole having wild-type rates of 95% and 99%, respectively.

**Table 1:** Minimum inhibitory concentration (MIC) and Minimum Effective Concentration (MEC) values for *Aspergillus* species (*n* = 236)

Species	Minimum inhibitory concentrations (mg/L) [*Minimum Effective Concentration]													MIC_50_	MIC_90_	CLSI ECV Interpretation (%WT)
	≤0.008	0.015	0.03	0.06	0.125	0.25	0.5	1	2	4	8	≥16	Total			
Amphotericin B (*n*=236)														**2**	**4**	
*Aspergillus *section Fumigati				2	3	10	15	24	63	**2**			119	2	2	98
*Aspergillus* section Flavi						1	1	9	18	7			36	2	4	100
*A. terreus *							2	5	15	10			32	2	4	100
*A. niger*				1	1	2	2	17	6				29	1	2	100
*Other Aspergillus spp.*						1	1	3	9	5	2		21	2	4	–
*Voriconazole (n=234)*														**0.25**	**1**	
*Aspergillus *section Fumigati				4	14	65	25	3	**4**	**1**	**1**		117	0.25	0.5	95
*Aspergillus *section Flavi						10	16	10					36	0.5	1	100
*A. terreus *				1	3	17	11						32	0.25	0.5	100
*A. niger*				1	2	4	16	5			1		29	0.5	1	100
*Other Aspergillus spp.*				2	3	2	2	5		4	3		21	1	8	–
*Posaconazole (n=217)*														**0.25**	**0.5**	
*Aspergillus *section Fumigati	2	4	4	4	47	39	7	3					110	0.125	0.25	–
*Aspergillus *section Flavi					6	21	6						33	0.25	0.5	100
*A. terreus *			1	3	19	6							29	0.125	0.25	100
*A. niger*		1		2		15	9						27	0.25	0.5	100
*Other Aspergillus spp.*		1		1	1	2	8	2	3			1	19	0.5	2	NB
*Itraconazole (n=236)*														**0.25**	**0.5**	
*Aspergillus *section Fumigati		2	2	3	8	58	40	5	**1**				119	0.25	0.5	99
*Aspergillus* section Flavi					2	28	6						36	0.25	0.5	100
A. terreus			2	1	9	20							32	0.25	0.25	100
*A. niger*				1		2	19	6	1				29	0.5	1	100
Other *Aspergillus* spp.			1		3	3	6	8					21	0.5	1	–
Isavuconazole (n=40)														**0.5**	**2**	
*Aspergillus* section Fumigati				1	1	4	15	3	1				24	0.5	1	–
*Aspergillus* section Flavi						1	1						2	–	–	–
*A. terreus *					1	1							3	–	–	–
*A. niger*								2	1		1		4	–	–	–
Other *Aspergillus* spp.							2	3		2			7	–	–	–
Caspofungin (n=38)*														**0.06**	**0.12**	
*Aspergillus* section Fumigati			3	8	5	1							17	0.06	0.12	100
*Aspergillus* section Flavi	1	3		3			1						8	–	–	100
*A. terreus *			2	1	1		**1**						5	–	–	80
*A. niger*			3	3									6	–	–	100
Other *Aspergillus* spp.				1	1								2	–	–	–
Micafungin (n=194)*														**0.015**	**0.03**	
*Aspergillus* section Fumigati	21	56	15										92	0.015	0.03	–
*Aspergillus* section Flavi	5	17	7				1						30	0.015	0.03	–
*A. terreus *	6	15	7										28	0.015	0.03	–
*A. niger*	7	6	12	2									27	0.03	0.03	–
Other *Aspergillus* spp.		9	5	2	2								18	0.03	0.125	–
Anidulafungin (n=194)*														**0.015**	**0.03**	
*Aspergillus* section Fumigati	1	75	16										92	0.015	0.03	–
*Aspergillus* section Flavi	2	22	5				1						30	0.015	0.03	–
*A. terreus *		17	11										28	0.015	0.03	–
*A. niger*		18	8	1									27	0.015	0.03	–
Other *Aspergillus* spp.		5	7	4	2								18	0.03	0.125	–

*ECV* Epidemiological Cut-off Value, *WT* Wild-type

*Minimum effective concentration (MEC) only applies to the echinocandin antifungals (anidulafungin, micafungin, caspofungin)

Echinocandins demonstrated low MIC values overall, with micafungin and anidulafungin both having an MIC_50/90_ of 0.015/0.03 mg/L on pooled *Aspergillus* spp.. Amphotericin B displayed intermediate activity against *Aspergillus* section Fumigati with approximately half of the isolates having an MIC ≥ 2 mg/L. A total of 15 cryptic species underwent susceptibility testing (Table 2). High triazole MIC values were observed among cryptic species with a voriconazole MIC_50/90_ of 2/8 mg/L.

**Table 2:** Antifungal MIC and MEC* values (mg/L) for cryptic *Aspergillus* species (*n* = 15)

Species	Year Collected	Voriconazole	Posaconazole	Itraconazole	Isavuconazole	Micafungin*	Anidulafungin*
*Aspergillus insuetus*	2018	4	1	0.5		0.06	0.06
*Aspergillus unguis*	2019	0.12	0.5	0.5		0.03	0.03
*Aspergillus ochraceus*	2019	0.5	0.5	0.5		0.03	0.125
*Aspergillus ustus*	2019	8	> 8	1		0.03	0.06
*Aspergillus wisconsinensis*	2019	0.5	0.5	0.25		0.015	0.015
*Aspergillus fischeri*	2019	2	1	1		0.015	0.015
*Aspergillus lentulus*	2020	4	1	2		0.015	0.015
*Aspergillus vericolor*	2020	0.25	0.5	1		0.015	0.015
*Aspergillus calidoustus*	2020	8	2	1		0.125	0.125
*Aspergillus calidoustus*	2021	4	2	1	1	0.03	0.015
*Aspergillus sydowii*	2021	1	0.5	0.5	0.5	0.015	0.015
*Aspergillus oryzae*	2021	0.25	0.25	0.25	0.5	0.015	0.03
*Aspergillus lentulus*	2021	8	0.5	1	4	0.015	0.03
*Aspergillus ochraceus*	2021	1	1	1	1	0.06	0.06
*Aspergillus calidoustus*	2021	4	2	1	1	0.015	0.03

**Fig. 1:**
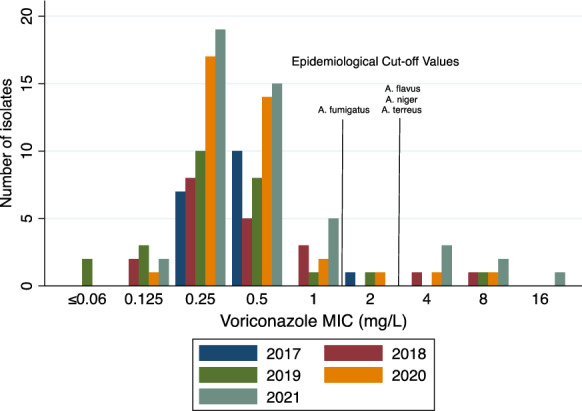
Voriconazole MIC values in *Aspergillus* species (*n* = 150) according to year (2017–2021) in Queensland, Australia

**Fig. 2:**
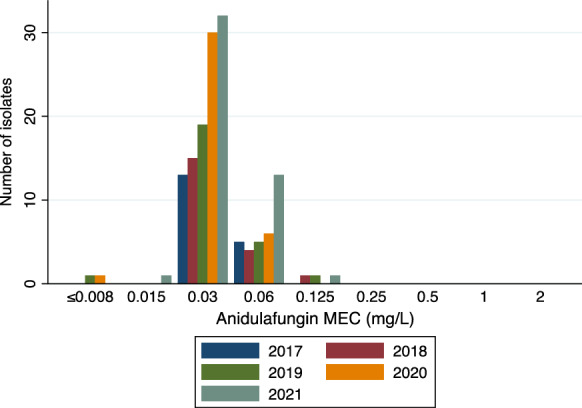
Anidulafungin MEC values in *Aspergillus* species (*n* = 150) according to year (2017–2021) in Queensland, Australia

**Fig. 3:**
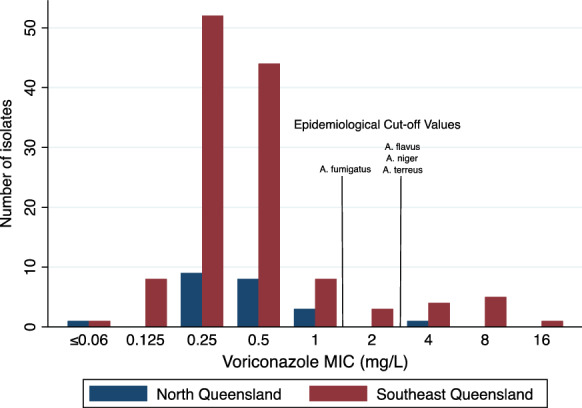
Voriconazole MIC values in *Aspergillus* species (*n* = 236) according to geographical location (North Queensland versus Southeast Queensland)

Voriconazole and anidulafungin MIC values of isolates obtained between 2017 and 2021 (*n* = 150) were analysed according to year (Figs. 1 and 2). In 2021, there were a total of 6 isolates (13%) with a voriconazole MIC value of ≥ 2 mg/L; this compared to a total of 3 (8%), 2 (8%), 2 (10%), and 1 (6%) identified in 2020, 2019, 2018 and 2017, respectively. Anidulafungin MIC values did not appear to change during this 5-year period with > 95% of isolates obtained between 2017 and 2021 having an MIC value between 0.03 and 0.06 mg/L. Thirty-seven isolates (15.7%) came from North Queensland; MIC distribution was not different between North and Southeast Queensland isolates (Fig. 3).

## Discussion

Here we describe the speciation and antifungal resistance profiles of 236 *Aspergillus* spp. causing invasive fungal disease in Queensland, Australia; analyses incorporating the year collected, geographic location, antifungal, and species (inc. cryptic) were undertaken. *Aspergillus* spp. identified, followed a similar frequency distribution to other datasets with the most frequently encountered species being *Aspergillus* section Fumigati [15]. More cryptic *Aspergillus* spp. were identified (*n* = 15, 6.4%) later in the study period, most likely contributed by the incorporation and increased use of molecular identification techniques. It is estimated that between 3 and 15% of IA infections are caused by cryptic species [16, 17]. Correct identification is paramount as some of the cryptic species, particularly *A. lentulus*, *A. alliaceus*, *A. sydowii*, *A. calidoustus*, *A. keveii*, *A. insuetus*, and *A. fumigatiaffinis*, demonstrate resistance to both azole and polyene antifungals [18, 19] as was demonstrated in this study where many cryptic isolates exhibited a voriconazole MIC between 4 and 8 mg/L.

Overall, our isolates displayed similar MIC values to those presented in other regions, both locally and worldwide [20] [21]. We noted that recovery of *Aspergillus* spp. with high voriconazole MIC values occurred more frequently later during the study period. Although, there was an overall increase in isolates being sent for AFST during this time. This is similar to the global trend of rising azole resistance [21–34]. A Dutch survey of unselected clinical *Aspergillus* section Fumigati isolates demonstrated an increase in resistance from 7% in 2014 to 15% in 2017 [35]. This is of particular concern given that azole resistance is associated with treatment failure [36–38].

Triazole resistance among clinically significant *Aspergillus* spp. is much higher in other regions around the world. A study from Turkey documented a 10.2% itraconazole resistance rate when they investigated 746 isolates collected from 1999 to 2012[24]. A similar study conducted in Japan noted the prevalence of azole-resistance *Aspergillus* spp. to be 8.3%[39]. A study which observed resistance rates among Latin American and African isolates noted triazole-resistance in 6.9% of samples in Mexico, 8.3% in Paraguay, 9.8% in Peru, and 2.2% in Nigeria [40].

There was no difference in voriconazole MIC values between *Aspergillus* spp. acquired in North Queensland when compared to Southeast Queensland, Australia, although the numbers from North Queensland were small. Climate and temperature change has been posed as a contributor to the emergence of different fungal pathogens and worsening antifungal resistance [41]. In addition, agricultural fungicides are also a known contributor to azole resistance among *Aspergillus* spp. Queensland is a large state with variations in climate and agriculture depending on the region. This gives added importance to the surveillance of antifungal resistance patterns over time across the whole state.

There are several limitations to this study which need to be outlined. Firstly, isolates included were obtained from public laboratories only and were from individuals with a high likelihood of invasive fungal disease. True epidemiological surveillance would incorporate testing of all *Aspergillus* spp. isolates from all laboratories and include those obtained from the environment. Secondly, there were no strict rules or criteria enforced with regards to triggering susceptibility testing of isolates. Clinical interpretation and decision making by the clinical microbiologist occurred. Thirdly, not all isolates were identified using molecular methods. In addition, amplicon sequencing targeting the ITS region was used for molecular identification which may misidentify some species of *Aspergillus* when used alone. Sequencing a portion of the beta-tubulin (*BenA*) and calmodulin (*CaM*) genes has been recommended for more high-resolution speciation [42]. Fourthly, there were changes to the methodology of antifungal susceptibility testing during the study period, including the antifungals tested. Our isolates were all tested at the same reference laboratory which prevented interlaboratory variability in results.

## Conclusion

IA carries a significant burden of disease globally, particularly among individuals with impaired immunity. Increasing reports of antifungal resistance among *Aspergillus* spp. is concerning and warrants further investigation both locally and worldwide. Reliable resistance data in *Aspergillus* spp. is difficult to obtain given that susceptibility testing is generally not a part of international antimicrobial resistance programs. Moreover, in Australia there is limited data on antifungal susceptibility patterns amongst clinically relevant mould species. Active surveillance of both the emergence of *Aspergillus* spp. and changes in antifungal susceptibility patterns over time is crucial to informing clinicians and treatment guidelines. Understanding the effects of the varied environmental pressures in different parts of Australia on clinically important *Aspergillus* spp. is also paramount.
